# A New Mechanism for Formation of Glycine from Glyoxylic Acid: the Aza‐Cannizzaro Reaction

**DOI:** 10.1002/chem.202403202

**Published:** 2024-11-06

**Authors:** Dean R. Jarois, Lars E. Schimmelpfennig, Samuel H. Gellman

**Affiliations:** ^1^ Department of Chemistry University of Wisconsin – Madison 1101 University Ave Madison WI 53706 United States

**Keywords:** Prebiotic chemistry, Glycine, Hydride, Aza-Cannizzaro reaction

## Abstract

Glyoxylic acid and glycine are widely considered to have been important prebiotic building blocks. Several mechanistic routes have been previously examined for conversion of glyoxylic acid to glycine. Here we provide evidence for a new mechanistic path. Glycine is spontaneously formed from glyoxylic acid in ammonium‐rich aqueous solutions at neutral pH; oxamic acid is generated as well. Hydride transfer from the glyoxylate‐derived hemiaminal to the corresponding iminium ion appears to underlie this transformation. This proposed mechanism parallels the well‐known Cannizzaro reaction mechanism, which leads us to suggest the designation “aza‐Cannizzaro reaction.” This discovery offers a new perspective on prebiotic nitrogen incorporation because glycine can be a source of nitrogen for more complex molecules, including other α‐amino acids.

## Graphical abstract

Glyoxylate imine is found to undergo Cannizzaro‐like disproportionation under mild conditions to form glycine. The mechanism by which an iminium‐hemiaminal pair disproportionate into an amide and amine has not been previously reported.

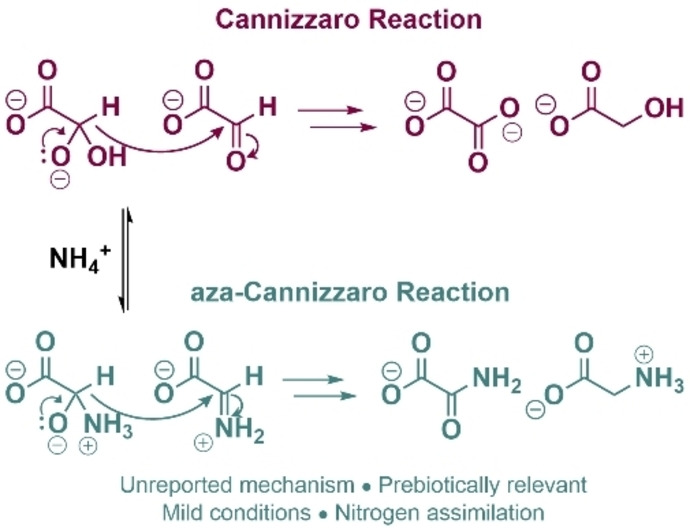

## Introduction

The emergence of life on Earth presumably required the availability of molecules identical or similar to those essential in contemporary biochemistry, including carbohydrates, α‐amino acids and nucleotides. Reaction cycles that might have occurred on Earth to generate such building blocks have been widely explored. Complementary efforts have identified potential building blocks in interstellar or circumstellar environments, from which these molecules might have been deposited on Earth. The specific collection of molecules that preceded the first life form cannot be known with certainty, nor can the processes that generated those compounds; nevertheless, it is valuable to characterize mechanistic routes that might have provided prebiotic building blocks. Even when paths to a likely building block have been identified, the discovery of new mechanistic routes enhances our perspective on prebiotic chemistry.

Glyoxylic acid was proposed by Eschenmoser as a key precursor for diverse prebiotic building blocks,^[1]^ and considerable evidence has been presented in support of this hypothesis.[^2^, ^3^] Glyoxylic acid was detected in recent extensions of the Miller‐Urey experiments,^[4]^ and Eckhardt et al. have demonstrated a plausible path to glyoxylic acid in interstellar ices.^[5]^ Glyoxylic acid can react to form a variety of potential prebiotic precursors; reaction with ammonia can generate glycine, which can subsequently undergo transamination with α‐ketoacids to generate other α‐amino acids.[^1^, ^6^, ^7^] Alternatively, glyoxylic acid can be generated from glycine via reaction with formaldehyde, with methylamine as by‐product.^[8]^ Glycine has been found in comets, but efforts to detect glycine in the interstellar medium have not been successful.[^9^, ^10^, ^11^] At this point, it is not clear whether glyoxylic acid should be regarded as a prebiotic precursor of glycine or vice versa; perhaps both transformations were important in different contexts.

Several mechanisms to generate glycine from glyoxylic acid and ammonia have been described previously. Glyoxylic acid and ammonia react with iron(II) salts to form glycine.^[12]^ This result was explained by proposing single electron transfer steps from Fe(II) to the imine formed by condensation of ammonia with glyoxylic acid. A related reductive amination process was suggested for formation of glycine from glyoxylic acid and ammonia in an aqueous sodium silicate solution.^[13]^ A different type of reductive amination mechanism has been proposed for reaction of glycine imine with glyoxylic acid.^[14]^ Glyoxylic acid and ammonium sulfate react at acidic pH to form N‐oxalylglycine.[^15^, ^16^, ^17^] This transformation, which was proposed to proceed via a transamination‐like mechanism, does not directly generate glycine.^[15]^ Strongly acidic conditions were required for subsequent hydrolysis of *N*‐oxalylglycine to liberate glycine. A comparable transformation was reported for ethyl glyoxylate and ammonium acetate in an organic solvent to generate the diethyl ester of *N*‐oxalylglycine; in this case, no further reaction to generate glycine was described.^[18]^ Reaction of glyoxylic acid with diamidophosphate (an ammonia source) and cyanide in pH 7 carbonate buffer leads to formation of glycine.[^19^, ^20^] This transformation was attributed to a Bucherer‐Bergs mechanism, which, in common with the Strecker mechanism, proceeds via an α‐amino nitrile intermediate.

We introduce a mechanism for formation of glycine from glyoxylic acid and ammonia at neutral pH that differs fundamentally from the precedents outlined above. The key step is an “aza‐Cannizzaro reaction,” which is analogous to the well‐known Cannizzaro reaction that generates a carboxylic acid and an alcohol from two aldehyde molecules. Possible roles for authentic Cannizzaro reactions in prebiotic processes have been examined,[^21^, ^22^] but the aza‐Cannizzaro mechanism we describe has not been considered.

## Results and Discussion

**Figure 1:**
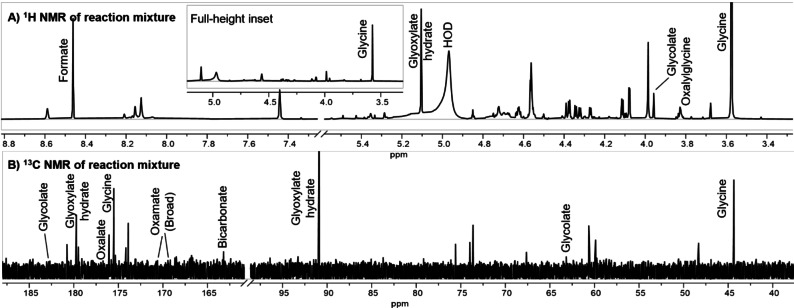
Glyoxylate is transformed to glycine when heated to 50 °C for 48 hr in ammonium‐rich buffers (0.5 M) at varying pH. Analysis of products by A) ^1^H‐presat NMR (selective pulse at 4.7 ppm to attenuate HOD signal) (500 MHz) with full‐size inset to show relative abundance of glycine; and B) ^13^C NMR (125 MHz, with 5 s relaxation delay to visualize all signals). Both spectra obtained from 0.5 M glyoxylate and 0.5 M ammonium phosphate that was neutralized to pH 7 and heated 48 hr at 50 °C. Aliquot was mixed with an equal volume of D_2_O and referenced with TSP (3‐(trimethylsilyl)propionic‐2,2,3,3‐d_4_ acid, sodium salt) prior to analysis (see supplementary information). Identifiable resonances are labelled.

Heating a solution of 0.5 M glyoxylic acid in 0.5 M ammonium phosphate buffer, pH 7, to 50 °C for 48 hr results in formation of multiple new species. Analysis of this mixture by ^1^H and ^13^C NMR allowed us to identify several of the products, the most prominent of which was glycine (Figure 1). Other products included oxamic acid, glycolic acid, oxalic acid and formic acid. Bicarbonate was also detected in some samples (see Figure S4 for more information). A small amount of *N*‐oxalylglycine was formed. Several unidentified ^1^H NMR resonances displayed splitting patterns that suggested carbon‐carbon bond formation. Quantitative ^1^H NMR analysis indicated that ~11 % of the starting glyoxylic acid in a 0.5 M starting solution was converted to glycine at pH 7. The glycine yield was slightly higher at elevated pH (up to 10); below pH 7, the glycine yield declined (Table 1).

**Table 1:** Glycine yield from 0.5 M glyoxylic acid under differing experimental conditions based on integration of ^1^H NMR signals relative to a TSP (3‐(trimethylsilyl)propionic‐2,2,3,3‐d_4_ acid, sodium salt) standard (note that maximum glycine yield is 50 %; see text).

Buffer/Salt	pH	%Glycine Yield
Ammonium Phosphate	5	2.0
Ammonium Phosphate	6	2.4
Ammonium Phosphate	7	10.7
Ammonium Phosphate	8	12.7
Ammonium Phosphate	9	12.1
Ammonium Phosphate	10	13.8
Ammonium Acetate	7	12.7
Ammonium Sulfate	7	15.4

**Figure 2:**
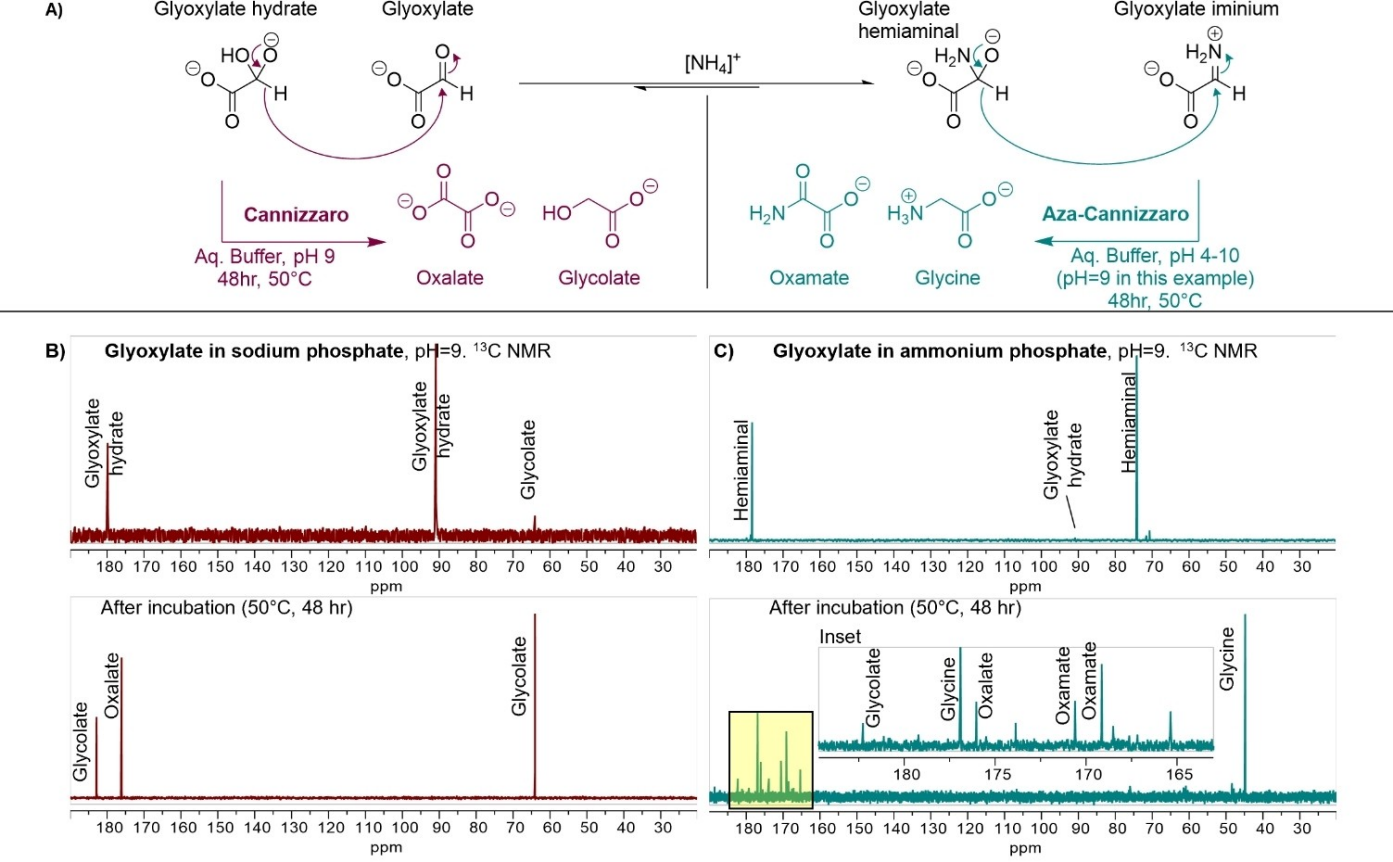
A) glyoxylate undergoes the well‐known Cannizzaro reaction at elevated pH. In the presence of ammonium, glyoxylate exists in equilibrium with the corresponding hemiaminal and iminium, which are proposed to disproportionate into glycine and oxamate via an “aza‐Cannizzaro” mechanism. B,C) The Cannizzaro transformation of glyoxylate into glycolate and oxalate and the aza‐Cannizzaro transformation into glycine and oxamate can be monitored by ^13^C NMR (125 MHz). Glyoxylate (0.5 M) was neutralized and incubated at 50 °C for 48 hr in pH 9 sodium phosphate buffer (0.5 M) or pH 9 ammonium phosphate (0.5 M) buffer to form the products indicated.

Glycine formation occurred to a similar extent when phosphate was replaced with acetate or sulfate in the pH 7 buffer (Table 1); however, no glycine was detected when glyoxylic acid was heated in deionized water with 1 eq. ammonium hydroxide. We suspect that the buffer anions serve as proton transfer catalysts in this reaction, a role that is well‐precedented for nucleophilic additions to carbonyl compounds in water.[^23^, ^24^] Glycine formation was considerably slower at room temperature relative to 50 °C. The presence or exclusion of atmospheric oxygen did not affect glycine formation. This process was very sensitive to the starting concentration of glyoxylic acid; below 0.25 M, very little reaction progress was observed after 48 hr at 50 °C. Carbon‐13 NMR analysis of a freshly prepared solution of glyoxylic acid in 0.5 M ammonium phosphate buffer, pH 7, revealed partial formation of the hemiaminal, a species that has been previously observed (Figure 2).[^25^, ^26^, ^27^] The extent of hemiaminal formation was increased when ammonium hydroxide was added. Dissolution of glyoxylic acid in water alone led to nearly complete conversion to the hydrate, but the hydrate was not the major species in the ammonium‐containing solutions. These observations are consistent with computational results from Mayer and Moran, who concluded that the hemiaminal is lower in energy than glyoxylate itself, the imine or the hydrate in aqueous solution.^[28]^

Based on our ^13^C NMR data and the reported computational results,^[28]^ we hypothesize that glycine formation in ammonium‐rich buffer occurs via a process analogous to the Cannizzaro reaction (Figure 2A). Authentic Cannizzaro reactions require alkaline conditions.[^29^, ^30^] Indeed, when a solution of glyoxylic acid in sodium phosphate buffer, pH 9, was heated to 40 °C for 21 hr, both oxalic acid and glycolic acid were detected via ^13^C NMR (Figure 2B), as expected for a standard Cannizzaro reaction. In ammonium phosphate buffer, pH 7, we propose that the hemiaminal derived from glyoxylic acid reacts with the iminium derived from glyoxylic acid to generate glycine and oxamic acid via hydride transfer. This hypothesis is consistent with our observation that formation of glycine is accompanied by formation of comparable amounts of oxamic acid. Small amounts of glycolic acid and oxalic acid were observed after 48 hr at 50 °C in ammonium phosphate buffer, pH 7, which suggests that the aza‐Cannizzaro reaction is accompanied by a low level of authentic Cannizzaro reactivity under these conditions. Even at pH 9, heating glyoxylic acid in 0.5 M ammonium phosphate led to formation of much more glycine and oxamic acid relative to glycolic acid and oxalic acid (Figure 2B). Thus, it appears that the aza‐Cannizzaro pathway is preferred under alkaline conditions, if the ammonium concentration is sufficient.

We speculate that a key intermediate in the aza‐Cannizzaro reaction is the glyoxylate iminium ion (Figure 2A), which should be considerably more electrophilic than the neutral imine. Although this iminium ion is probably not the major species at pH>7, the anticipated reactivity of the iminium could nevertheless allow the transformation to proceed. At lower pH, the hemiaminal oxygen is likely to be protonated, which would presumably attenuate hydride donor reactivity. This O‐protonation could explain the decline in aza‐Cannizzaro reactivity at low pH (conditions under which the iminium should be abundant).

The small amount of N‐oxalylglycine we observed at pH 7 suggests that the process described by Yanagawa et al. occurs to a limited extent under our reaction conditions.^[15]^ However, three considerations indicate that this process cannot explain the formation of glycine itself under our conditions. (1) Yanagawa et al. reported that hydrolysis of N‐oxalylglycine to liberate glycine required 6 N HCl.^[15]^ (2) We observed that N‐oxalylglycine is stable to hydrolysis in 0.5 M ammonium phosphate buffer at 50 °C over 5 days. (3) Yanagawa et al. reported that N‐oxalylglycine formation was substantially enhanced at pH 4 relative to pH 8,^[15]^ which contrasts with our observation that glycine formation in 0.5 M ammonium phosphate buffer is diminished at pH≤6 relative to pH≥7 (Table 1).

**Figure 3:**
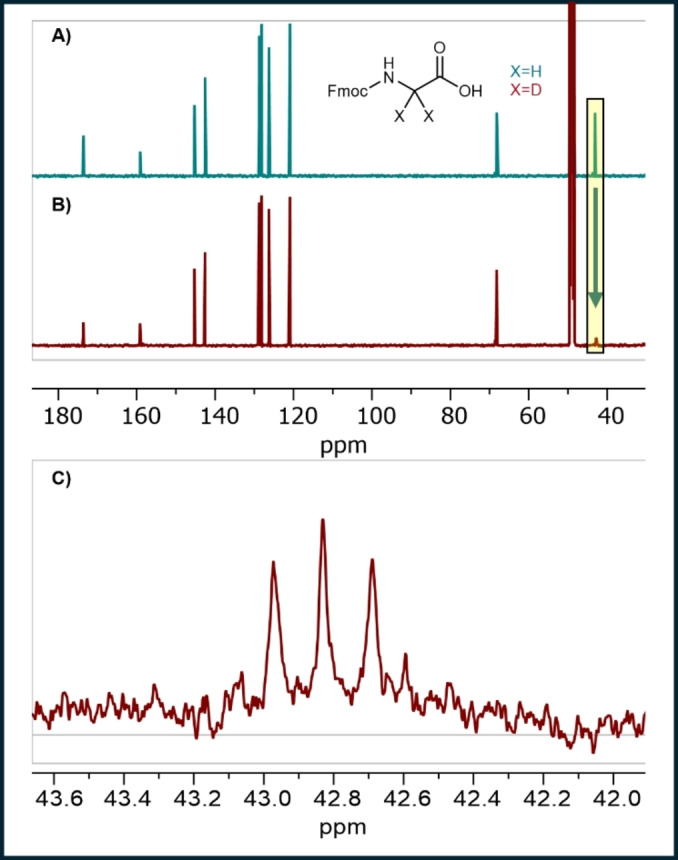
Representative ^13^C NMR (200 MHz) spectra. A) commercial Fmoc‐Gly‐OH in methanol‐d_4_. B) Fmoc‐Gly‐OH isolated from a reaction mixture that was generated from glyoxylic acid‐d. Note attenuation of the α‐carbon signal in B relative to A, which is consistent with the predicted lack of nOe enhancement because no H atoms are attached to this carbon. C) Expansion of the ^13^C NMR signal near 42.8 ppm for Fmoc‐Gly‐OH formed from glyoxylic acid‐d. This signal appears to be split into a quintet, as expected if there are two D atoms attached.

We undertook an isotopic labeling study to test the proposed aza‐Cannizzaro mechanism for glycine formation. Glyoxylic acid‐d was generated by reduction of oxalic acid with magnesium in deuterium oxide.^[31]^ The reaction mixture was acidified with phosphoric acid and then filtered to remove insoluble magnesium salts. The filtrate was mixed with ammonium phosphate monobasic and neutralized with ammonium hydroxide to pH 7 before heating to 50 °C for 48 hr. Deuterium NMR analysis of the reaction mixture revealed a signal at δ 3.63 ppm, which is consistent with formation of glycine‐d_2_. The reaction mixture was lyophilized, and the residue was treated with *N*‐(9‐fluorenylmethoxycarbonyloxy)succinimide in pyridine. Fmoc‐glycine was isolated from this reaction mixture and compared with a commercial sample of Fmoc‐glycine via proton‐decoupled ^13^C NMR (Figure 3). The two spectra are nearly identical; they differ only in the signal at 41.6 ppm. For commercial Fmoc‐glycine, the strong signal at 41.6 ppm arises from the α‐methylene carbon. For the sample derived from glyoxylic acid‐d, the corresponding signal centered at 42.8 ppm is much smaller and appears to be split into the quintet expected from coupling to two attached deuterium atoms. Overall, the NMR study supports the aza‐Cannizzaro mechanistic hypothesis by showing that both α‐hydrogens in glycine formed from glyoxylic acid in ammonium‐rich buffer originate from glyoxylic acid.

Formation of glycine from glyoxylic acid, an ammonium source and cyanide has been reported to occur via a Bucherer‐Bergs mechanism.[^19^, ^20^] We observed that inclusion of cyanide in a solution containing glyoxylic acid and ammonium phosphate suppressed glycine formation. We hypothesize that cyanohydrin formation is more favorable than hemiaminal formation, which would explain why cyanide inhibits the aza‐Cannizzaro pathway. This conclusion is consistent with a previous report that cyanide inhibits the reduction of 2‐oxoacids by cyanoborohydride.^[28]^

We note that Sud et al. used the designation “aza‐Cannizzaro” to describe the formation of diethyl *N*‐oxalylglycine from ethyl glyoxylate; however, this designation seems inappropriate for the reactivity described by these authors. The intramolecular analogue of the Cannizzaro mechanism would correspond to a 1,3‐sigmatropic hydrogen shift, which is predicted to be forbidden based on orbital symmetry.^[30]^ Indeed, computational results reported by Sud et al. indicated that the 1,3 shift would be far less favorable than a 1,2 shift followed by a 1,4 proton transfer. These authors considered neither an intermolecular aza‐Cannizzaro mechanism,^[18]^ nor the transamination‐like mechanism proposed by Yanagawa et al.^[15]^

## Conclusions

We have shown that glycine forms spontaneously from glyoxylic acid in an ammonium‐rich aqueous solution at neutral pH. This process is plausible as a prebiotic source of glycine, because glyoxylic acid has been widely discussed as a prebiotic precursor,[^2^, ^3^, ^6^, ^32^, ^33^] and ammonium‐containing minerals are found abundantly on Earth.^[34]^ Moderate heating was required to generate glycine on the laboratory timescale in our studies, but it seems reasonable to suggest that the aza‐Cannizzaro process could generate significant amounts of glycine at lower temperatures over longer periods. This glycine‐forming reaction is very sensitive to the concentrations of glyoxylic acid and ammonium; therefore, a prebiotic role might have required precursor concentration via evaporation. Abiotic availability of glycine could have been an important initiating step on the path toward the emergence of life since transamination reactions involving glycine and α‐keto acids can generate other α‐amino acids.[^6^, ^35^] Because transamination of α‐ketoacids with glycine regenerates one equivalent of glyoxylate, one can imagine linked prebiotic cycles allowing the assimilation of nitrogen, from ammonium, into organic compounds necessary for the origin of life.^[36]^

## Conflict of Interests

The authors declare no conflict of interest.

1

## Supporting information

As a service to our authors and readers, this journal provides supporting information supplied by the authors. Such materials are peer reviewed and may be re‐organized for online delivery, but are not copy‐edited or typeset. Technical support issues arising from supporting information (other than missing files) should be addressed to the authors.

Supporting Information

## Data Availability

The data that support the findings of this study are available in the supplementary material of this article.
