# Supplementary material for: A New Mechanism for Formation of Glycine from Glyoxylic Acid: the Aza‐Cannizzaro Reaction
Source: Chemistry. 2024 Nov 6;30(71):e202403202. doi: 10.1002/chem.202403202 (PMC11653230; doi:10.1002/chem.202403202)
Supplement: Supplementary file 1 — Supporting Information [file CHEM-30-e202403202-s001.pdf]

Chemistry–A European Journal

Supporting Information

A New Mechanism for Formation of Glycine from Glyoxylic Acid: the Aza-Cannizzaro Reaction

Dean R. Jarois, Lars E. Schimmelpfennig, and Samuel H. Gellman*

Supplementary Information

A New Mechanism for Formation of Glycine from Glyoxylic acid: the Aza-Cannizzaro Reaction

Dean R. Jarois, Lars E. Schimmelpfennig, Samuel H. Gellman*

Department of Chemistry, University of Wisconsin—Madison,

1101 University Ave, Madison, WI 53706 USA

Correspondence: gellman@chem.wisc.edu

Table of Contents:

Figure Title	Page
**Experimental Section**	**3**
**Figure S1.** Identification of glycine in the aza-Cannizzaro reaction mixture.	**5**
**Figure S2.** Identification of oxamic acid in the aza-Cannizzaro reaction mixture.	**6**
**Figure S3.** Identification of side products in the aza-Cannizzaro reaction mixture.	**7**
**Figure S4.** Identification of (bi)carbonate in aza-Cannizzaro reaction mixture.	**8**
**Figure S5.** Aza-Cannizzaro reaction occurs in the presence of multiple buffer anions.	**9**
**Figure S6.** Lack of reactivity observed in absence of counterion.	**10**
**Figure S7.** Aza-Cannizzaro reaction proceeds slowly at room temperature.	**11**
**Figure S8.** Atmospheric oxygen has no effect on aza-Cannizzaro reaction.	**12**
**Figure S9.** Aza-Cannizzaro reaction sensitive to starting concentration of glyoxylate.	**13**
**Figure S10.** Second-order glyoxylate consumption observed in aza-Cannizzaro reaction.	**14**
**Figure S11.** Aza-Cannizzaro reactivity observed over wide pH range.	**15**
**Figure S12.** Addition of ammonium drives hemiaminal equilibrium.	**16**
**Figure S13.** Comparable amount of glycine and oxamic acid in aza-Cannizzaro mixture.	**17**
**Figure S14.** Oxalylglycine stable to hydrolysis in ammonium phosphate buffer.	**18**
**Figure S15.** Spectra of glyoxylate-*d*.	**19**
**Figure S16.** Mass spectrum of Fmoc-glycine-d_x	**20**
**Figure S17.** Presence of cyanide inhibits aza-Cannizzaro reactivity.	**21**

Experimental Section

Water was purified to 18.2 MΩ with a Thermo Barnstead Nanopure system. Reagents were purchased from the following manufacturers: glyoxylic acid (50% in water, AlfaAesar), glyoxylic acid monohydrate (98%, Thermo), ammonium phosphate dibasic (reagent grade, Ward's), ammonium phosphate monobasic (lab grade, Ward's), ammonium hydroxide (30%, ACS grade, Sigma Aldrich), *N*-oxalylglycine (>98%, Santa Cruz Biotechnologies), glycine (electrophoresis grade, IBI), glycolic acid (ACS, Sigma Aldrich), oxalic acid anhydrous (98%, Thermo), oxamic acid (>95%, TCI), FmocOSu (ChemImpex), deuterium oxide (99.8%, Acros Organics), magnesium (ACS, Sigma Aldrich), sodium phosphate monobasic (BioXtra, Sigma Aldrich), sodium phosphate dibasic (ACS, Sigma Aldrich), ammonium acetate (molecular biology grade, Sigma Aldrich), ammonium sulfate (molecular biology grade, Sigma Aldrich), potassium cyanide (ACS, Sigma Aldrich), pyridine (ACS, Sigma Aldrich), methanol, (HPLC, Fisher), and phosphoric acid (85%, Sigma Aldrich).

NMR spectra were collected on a Bruker Neo 500 MHz spectrometer with a Prodigy cryoprobe, or, for measurements with deuterium-labelled samples, on a Bruker Avance 600 MHz spectrometer with a TCI-F cryoprobe. NMR samples were prepared by mixing equal volumes of the reaction mixture and D_2O. 3-(Trimethylsilylpropionic-2,2,3,3-d_4 acid sodium salt ("TSP," 98%, Sigma Aldrich) was used as a chemical shift reference. In quantitative studies, TSP was added prior to the reaction and used as an integration standard to account for possible evaporation or dilution when adding ammonium hydroxide. Preparative HPLC was performed on an Agilent 1260 Infinity II instrument with a Waters X-Select C18 CSH column (19x250 mm) with MeCN/water+0.1%TFA mobile phase. pH measurements were acquired on a Mettler AB15 instrument. Lyophilization was carried out on a Labconco FreeZone instrument. Reactions were carried out in a New Brunswick Scientific temperature-controlled shaker.

Conversion of glyoxylate to glycine and oxamic acid (the aza-Cannizarro Reaction). Glyoxylic acid monohydrate (230 mg, 2.5 mmol, 0.5 M, 1eq) was dissolved in 5 mL aqueous ammonium buffer (pH=7, 0.5 M ammonium phosphate, unless otherwise specified). Ammonium hydroxide (30% in water) was added dropwise until the pH returned to 7.0. The reaction vessel was capped and incubated for 48 hr at 50°C to produce glycine (10% conversion), oxamate (10%), and other products.

The Cannizzaro Reaction of glyoxylate. Glyoxylic acid monohydrate (230 mg, 2.5 mmol, 0.5 M, 1 eq) was dissolved in 5 mL aqueous sodium phosphate buffer (pH 9, 0.5 M sodium phosphate). Sodium hydroxide (10 M in water) was added dropwise until the pH returned to 9.0. The reaction vessel was capped and incubated for 48 hr at 50°C to produce glycolate and oxalate.

Preparation of glyoxylic acid-d. Oxalic acid anhydrous (4.50 g, 0.050 mol) was mixed with 10 mL deuterium oxide, and the solution was concentrated *in vacuo*. This procedure was repeated three times to provide oxalic acid-d_2. Meanwhile magnesium metal (5.00 g, 206 mmol, 4.1 eq) was added to 20 mL deuterium oxide. The mixture was cooled in an ice bath. A steady stream of dry nitrogen gas was directed into the flask. The oxalic acid-d_2 was dissolved in 50 mL D_2O, and this solution was added dropwise to the stirring magnesium slurry. The reaction vessel remained open to air to allow D_2 gas to escape. After the bubbling ceased, 6.7 mL phosphoric acid (85%) was added, and the solution was filtered to remove insoluble magnesium salts. The volume was corrected to 100 mL with nanopure water. If conversion were complete, this procedure would

generate a 0.5 M glyoxylic acid-d solution. The actual concentration was estimated by ^{13}C NMR and typically found to be less than 0.2 M. The solution was used without further purification.

Conversion of glyoxylic acid-d to Fmoc-glycine-OH-d_2. 100 mL of the glyoxylic acid-d solution was mixed with 5.72 g ammonium phosphate monobasic (0.048 mol), and ammonium hydroxide (30%) was added dropwise to reach pH 7.0. This solution was incubated for 48 hr at 50°C; the solution was then frozen and lyophilized. The powder was mixed with 100 pyridine, 15.3 g FmocOSu (0.050 mol) was added, and the mixtures were heated for 24 hr at 50°C. After cooling, the solution was filtered, and the filtrate was concentrated *in vacuo*. The dried residue was combined with 10 mL methanol, and this mixture was concentrated to 2 mL, which was passed through a 0.22 µm filter. Fmoc-Gly-d_2 was isolated via reverse-phase HPLC. The isolated fractions were concentrated *in vacuo* and analyzed with NMR in DMSO-d_6.

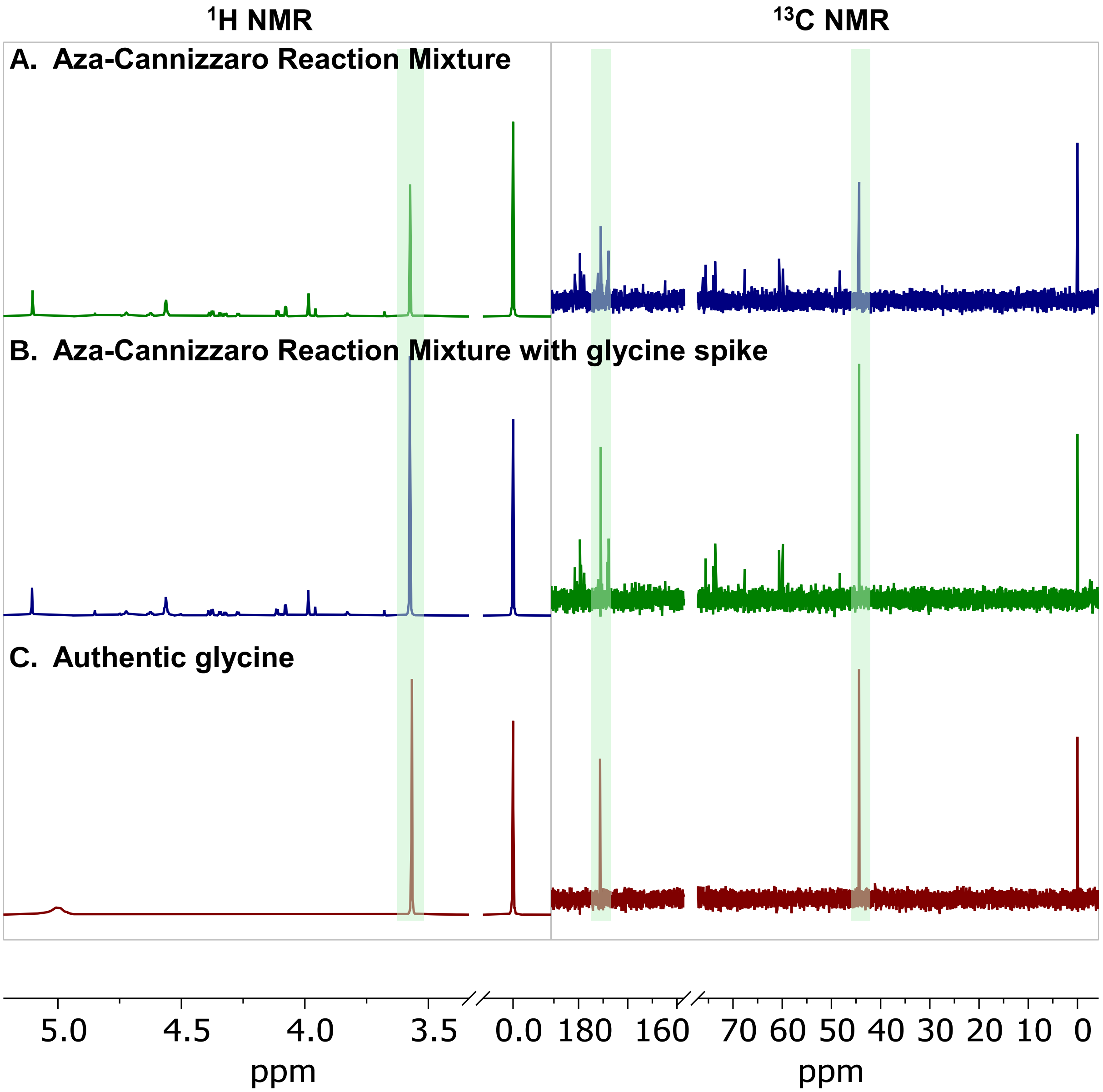

Figure S1. Identification of glycine in the aza-Cannizzaro reaction mixture. The presence of glycine in the aza-Cannizzaro reaction mixture was established by NMR, with 3.8 mM TSP (3-(trimethylsilyl)propionic-2,2,3,3-d_4 acid, sodium salt) as an internal standard. (A) Selected portions of ^{1}H NMR (selective pulse at 4.7 ppm to attenuate HOD resonance) (500 MHz; pulse sequence=zgpr30; relaxation delay=15s) and ^{13}C NMR spectra (125 MHz, zgpg30; relaxation delay=5s) for glyoxylate (500 mM) in pH 7 ammonium phosphate (500 mM) after 48 hr incubation at 50°C; the reaction solution was mixed with an equal volume of D_2O before analysis. (B) Selected portions of ^{1}H and ^{13}C NMR spectra of the same sample after addition of 23 mM glycine. (C) Selected portions of ^{1}H and ^{13}C NMR spectra of 23 mM glycine in 250 mM ammonium phosphate in 1:1 D_2O:H_2O.

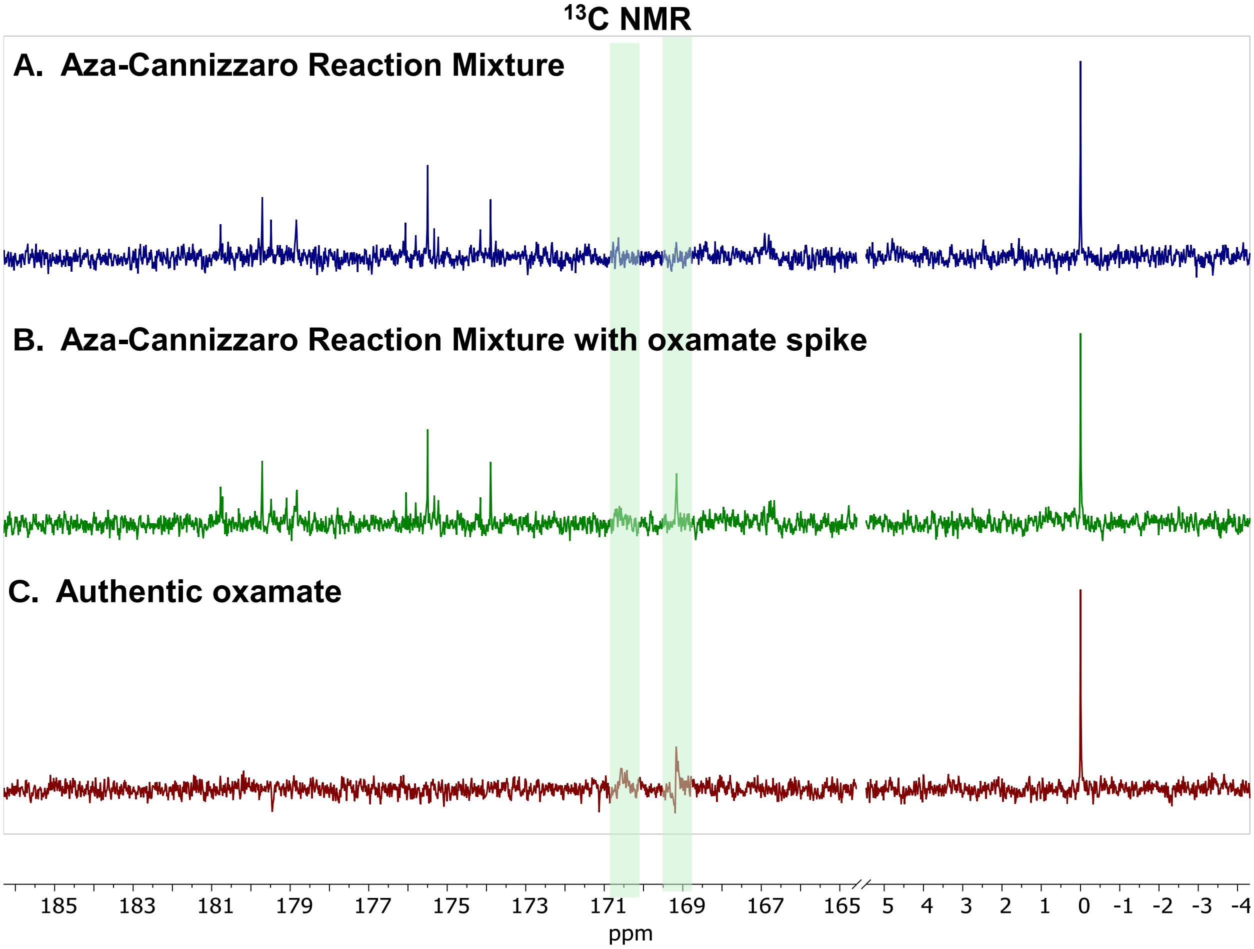

Figure S2. Identification of oxamic acid in the aza-Cannizzaro reaction mixture. The presence of oxamate in the aza-Cannizzaro reaction mixture was established by ^{13}C NMR, with 3.8 mM TSP (3-(trimethylsilyl)propionic-2,2,3,3-d$_4$ acid, sodium salt) as an internal standard. (A) Selected portions of the ^{13}C NMR spectrum (125 MHz, zgpg30; relaxation delay=5s) for glyoxylate (500 mM) in pH 7 ammonium phosphate (500 mM) after 48 hr incubation at 50°C; the reaction solution was mixed with an equal volume of D_2O before analysis . (B) Selected portions of the ^{13}C NMR spectrum of the same sample after addition of 23 mM oxamate. (C) Selected portions of ^{3}C NMR spectrum of 23 mM oxamate in 250 mM ammonium phosphate in 1:1 D_2O:H_2O. Note that oxamate signals are significantly broadened at pH 7 and require long relaxation times. See Figure S13.

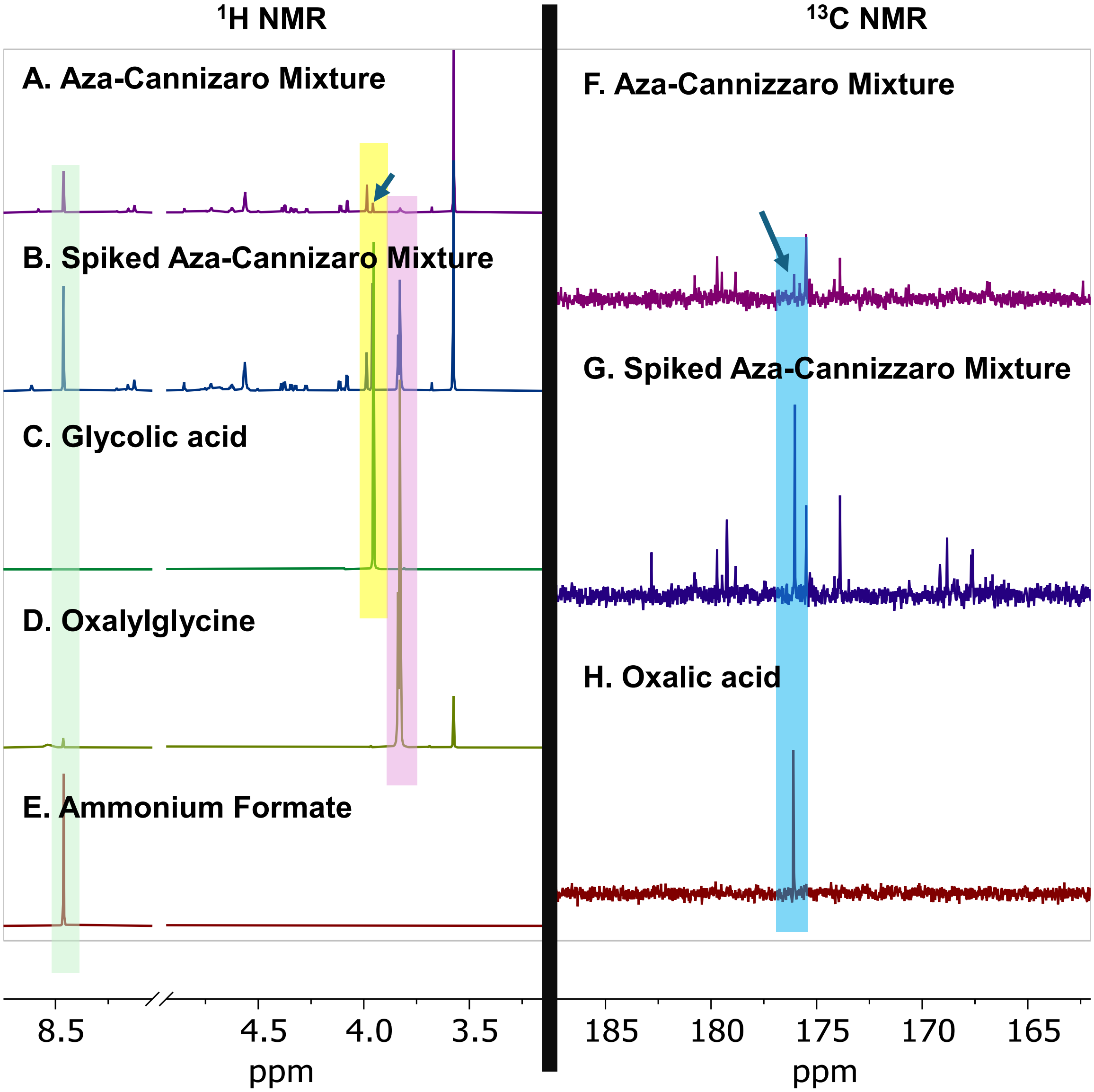

Figure S3. Identification of side products in the aza-Cannizzaro reaction mixture. The presence of glycolic acid, oxalylglycine, formate, and oxalate in the aza-Cannizzaro reaction mixture were established by NMR with 3.8 mM TSP (3-(trimethylsilyl)propionic-2,2,3,3-d_4 acid, sodium salt) as an internal standard. (A) and (F) represent ^{1}H-presat (selective pulse at 4.7 ppm to attenuate HOD resonance) (500 MHz; pulse sequence=zgpr30; relaxation delay=15s) and ^{13}C NMR spectra (125 MHz, zgpg30; relaxation delay=5s) for glyoxylate (500 mM) in pH=7 ammonium phosphate (500 mM) after 48 hr incubation at 50°C; the reaction was mixed with an equal volume of D_2O. (B) and (G) show the same sample spiked with 23 mM each of glycolic acid, oxalylglycine, ammonium formate, and oxalic acid. Note the highlighted signals. (C-E) and (H) are pure 23 mM samples of the nominal compound in 250 mM ammonium phosphate in 1:1 D_2O:H_2O. Glycolate and oxalate are present in very small amounts in the original sample and the corresponding resonances in (A) and (F) (respectively) are indicated with arrows.

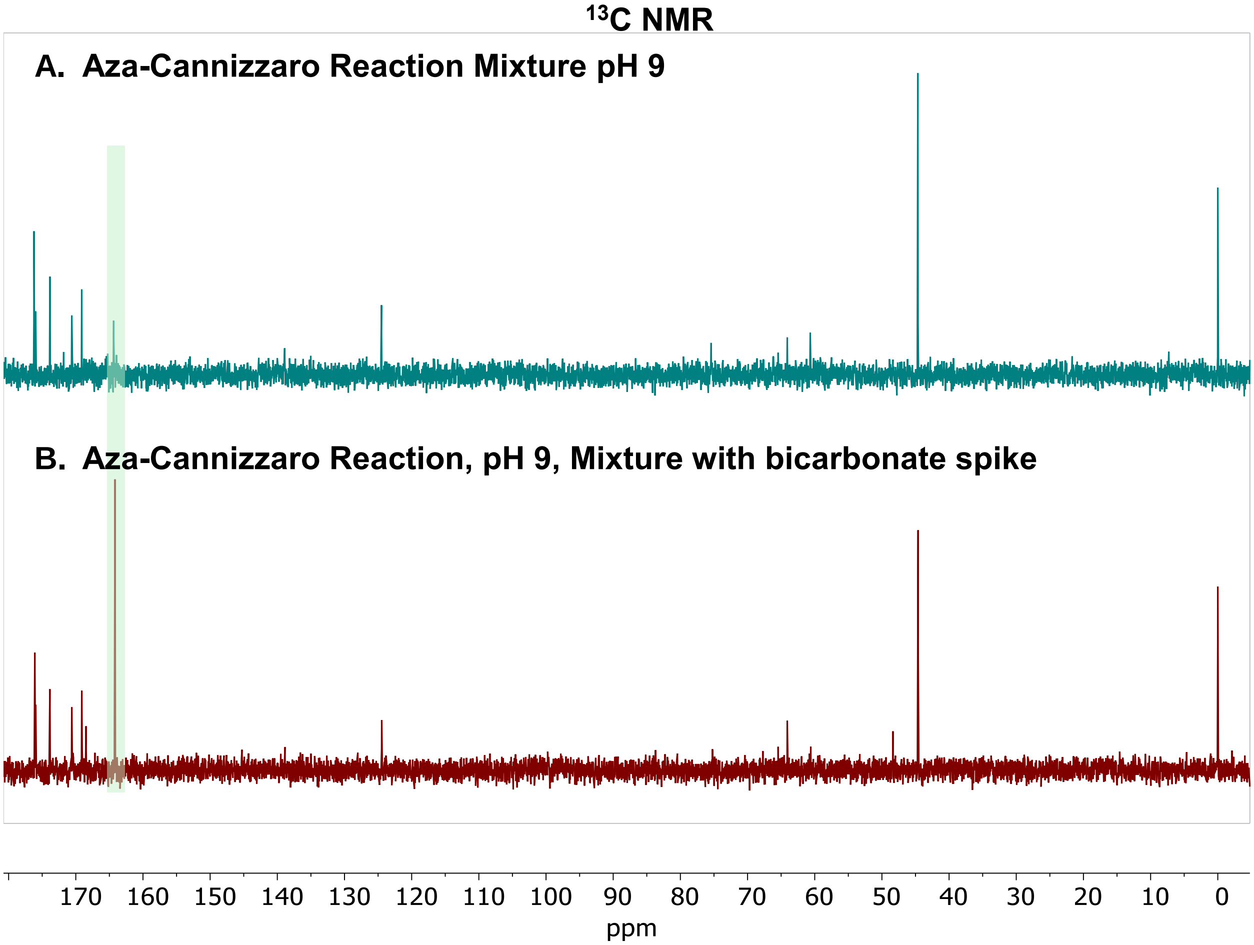

Figure S4. Identification of (bi)carbonate in the aza-Cannizzaro reaction mixture. (Bi)carbonate is observed in some, but not all, ^{13}C spectra of aza-Cannizzaro reaction mixtures we obtained. Under acidic or neutral conditions, (bi)carbonate is either not observed or in low abundance, presumably because of escaping CO_2 gas. Under basic conditions, the (bi)carbonate remains trapped in solution, such as in this pH 9 example with TSP (3-(trimethylsilyl)propionic-2,2,3,3-d_4 acid, sodium salt) as an internal standard. (A) The ^{13}C NMR spectrum (125 MHz, zgpg30; relaxation delay=2s) for glyoxylate (500 mM) in pH 9 ammonium phosphate (500 mM) after 48 hr incubation at 50°C; 600 µL reaction solution was mixed with 100 µL of D_2O before analysis. (B) The ^{13}C NMR spectrum of the same sample after addition of 75 µL saturated sodium bicarbonate. The (bi)carbonate resonance is highlighted in green.

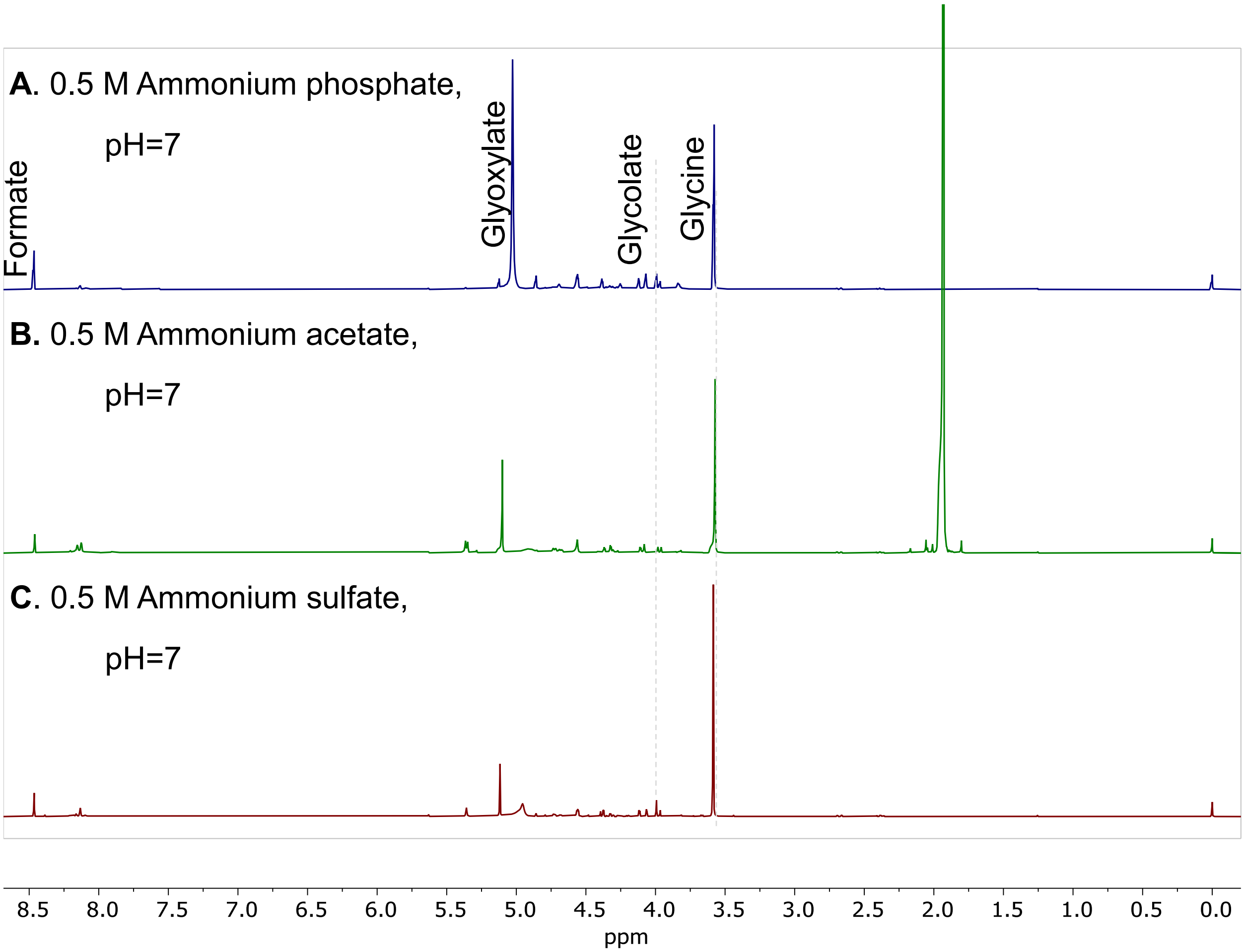

Figure S5. The aza-Cannizzaro reaction occurs in the presence of multiple buffer anions. ^{1}H-presat (selective pulse at 4.7 ppm to attenuate HOD resonance) (500 MHz; pulse sequence=zgpr30, with TSP (3-(trimethylsilyl)propionic-2,2,3,3-d_4 acid, sodium salt) standard) NMR spectra of glyoxylate (500 mM) in pH=7 ammonium solution (500 mM) after 48 hr incubation at 50°C; aliquot of reaction was mixed with an equal volume of D_2O before analysis. Note that glycine formation occurs in (A) ammonium phosphate, (B), ammonium acetate, and (C) ammonium sulfate.

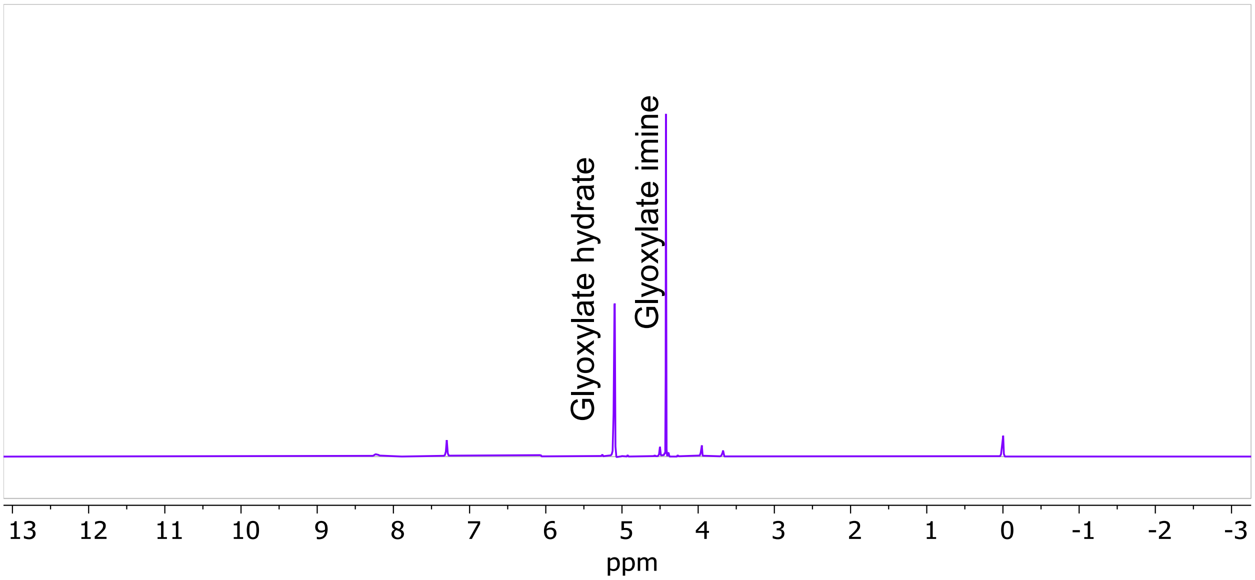

Figure S6. The aza-Cannizzaro reaction is impaired in the absence of phosphate, sulfate, or acetate. Glyoxylate (500 mM) was neutralized with ammonium hydroxide to pH 7 and incubated at 50°C for 48 hr. Reaction aliquot mixed with an equal volume of D_2O prior to analysis. Only small amounts of Cannizzaro and aza-Cannizzaro products were detected via ^1H NMR (selective pulse at 4.7 ppm to attenuate HOD resonance) (500 MHz; pulse sequence=zgpr30, TSP (3-(trimethylsilyl)propionic-2,2,3,3-d_4 acid, sodium salt) standard).

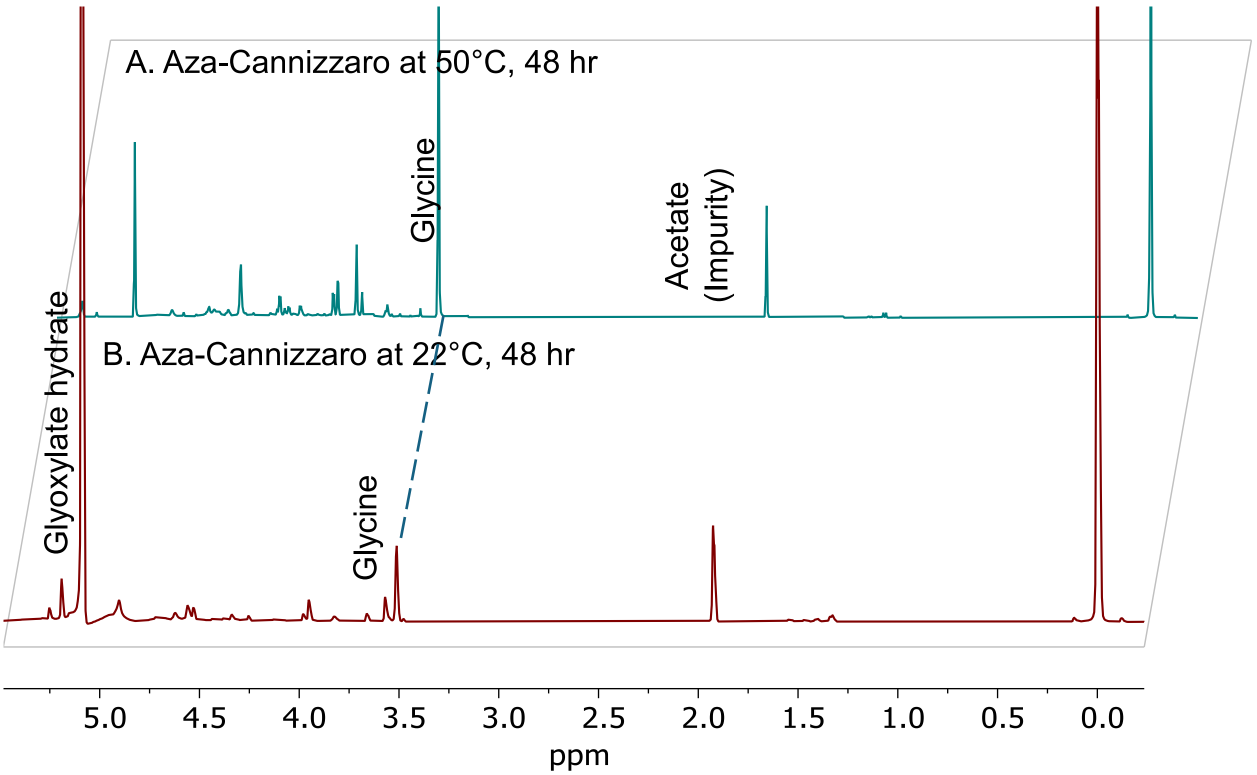

Figure S7. The aza-Cannizzaro reaction proceeds slowly at room temperature. Glyoxylate (500 mM) in ammonium phosphate (500 mM, pH 7) was either (A) incubated at 50°C for 48 hr or (B) at room temperature for 48 hr. Reaction aliquot combined with an equal volume of D_2O prior to analysis. Glycine formation is observed in both samples via ^{1}H-presat (selective pulse at 4.7 ppm to attenuate HOD resonance) NMR (500 MHz; pulse sequence=zgpr30, TSP (3-(trimethylsilyl)propionic-2,2,3,3-d_4 acid, sodium salt) standard), but to a lesser extent in the room temperature sample. Note the abundance of residual starting material at room temperature.

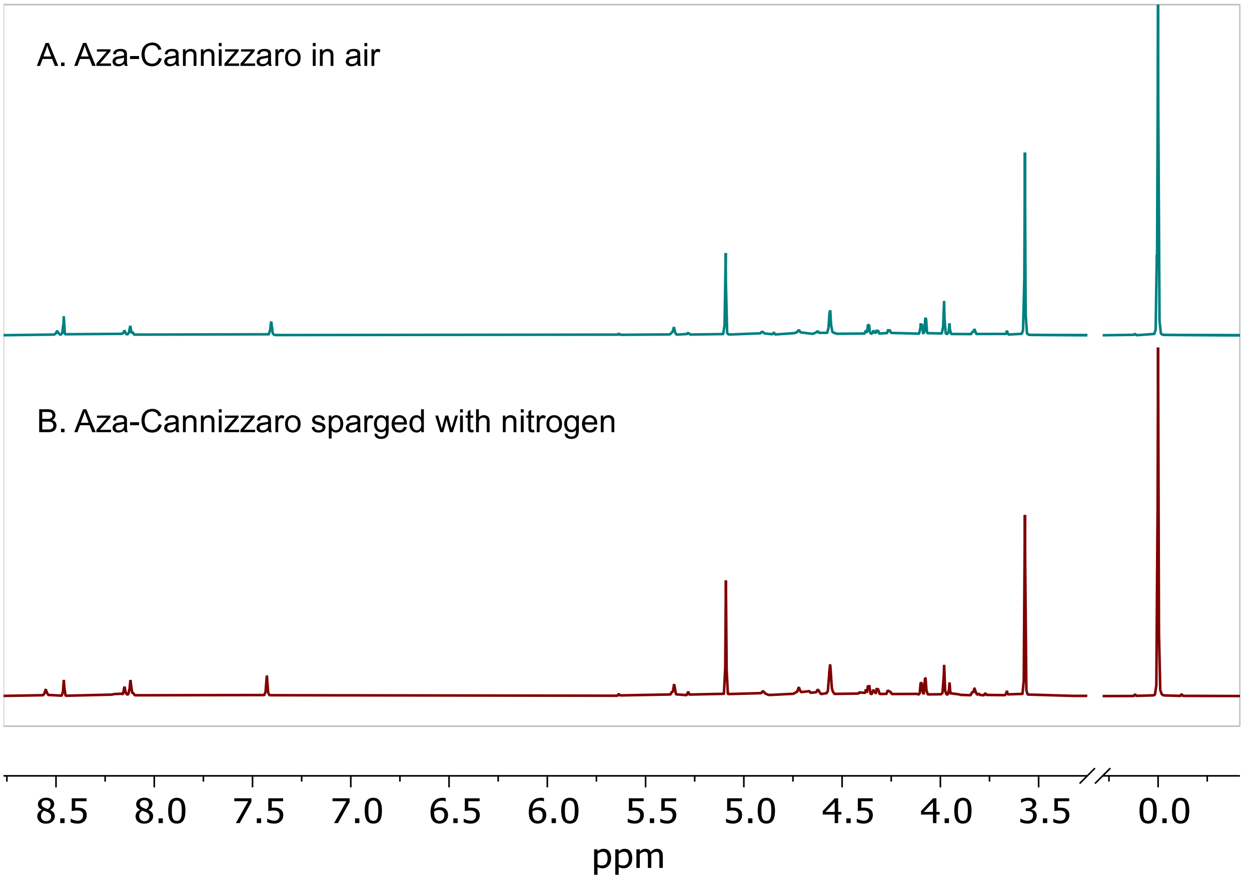

Figure S8. Atmospheric oxygen has no effect on the aza-Cannizzaro reaction. A solution containing glyoxylate (500 mM) in ammonium phosphate (500 mM, pH 7) was either (A) shaken to introduce oxygen or (B) sparged with nitrogen and incubated at 50°C for 48 hr. Reaction aliquot combined with an equal volume of D_2O prior to analysis. Glycine formation was observed in both samples via ^1H NMR-presat (selective pulse at 4.7 ppm to attenuate HOD resonance) (500 MHz; pulse sequence=zgpr30, TSP (3-(trimethylsilyl)propionic-2,2,3,3-d_4 acid, sodium salt) standard) with negligible observable differences between the two conditions.

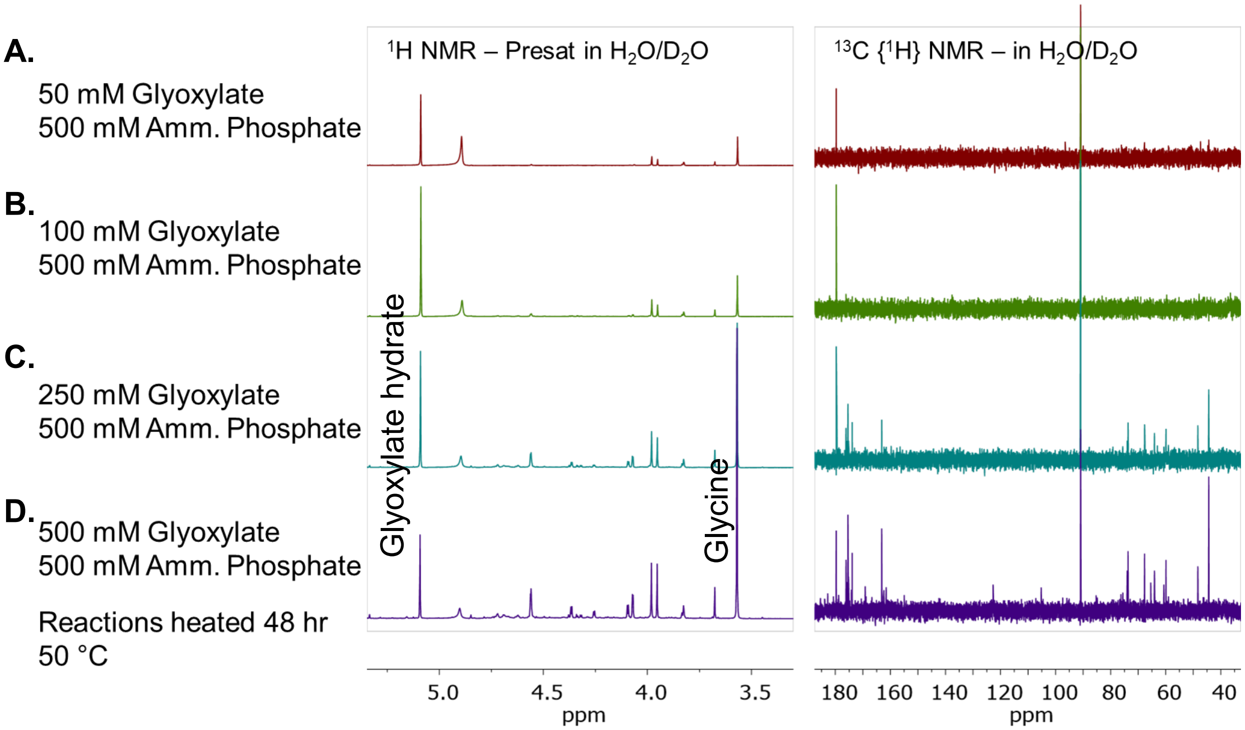

Figure S9. The aza-Cannizzaro reaction is sensitive to the starting concentration of glyoxylate. Glyoxylate at (A) 50 mM, (B), 100 mM, (C), 250 mM, or (D) 500 mM in ammonium phosphate (500 mM, pH 7) was incubated at 50°C for 48 hr. Reaction aliquot combined with an equal volume of D_2O prior to analysis. Glycine formation was observed in all samples via ^{1}H NMR-presat (selective pulse at 4.7 ppm to attenuate HOD resonance) (500 MHz; pulse sequence=zgpr30, TSP (3-(trimethylsilyl)propionic-2,2,3,3-d_4 acid, sodium salt) standard) and ^{13}C NMR spectroscopy (125 MHz, zgpg30; relaxation delay=2s). Starting material was also observed in each sample.

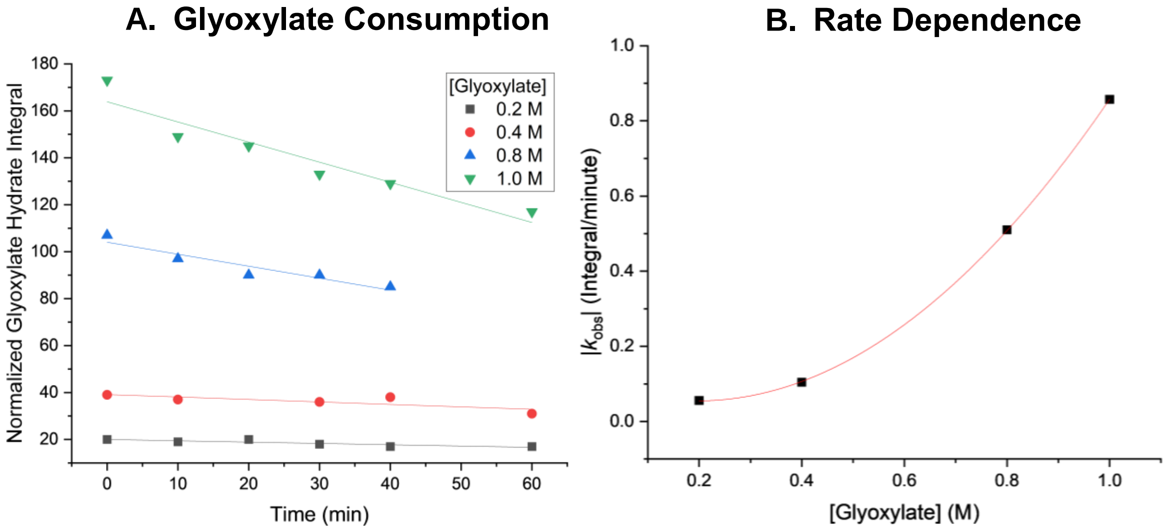

Figure S10. Second-order glyoxylate consumption under aza-Cannizzaro reaction conditions. Dependence of initial rate of glyoxylate consumption on [glyoxylate] in the aza-Cannizzaro reaction at pH 7 and 50°C. Aliquots were acquired throughout the first hour and spiked with a constant concentration of 3-(trimethylsilyl)propionic acid-d_4 as an NMR standard. A) The normalized glyoxylate hydrate integral (δ5.10 ppm) plotted as a function of time. B) The rate of glyoxylate consumption exhibits a second order dependence on concentration (2nd order polynomial fit; $y=0.10258-0.48801x+1.24354x^2$) $R^2=0.99998$).

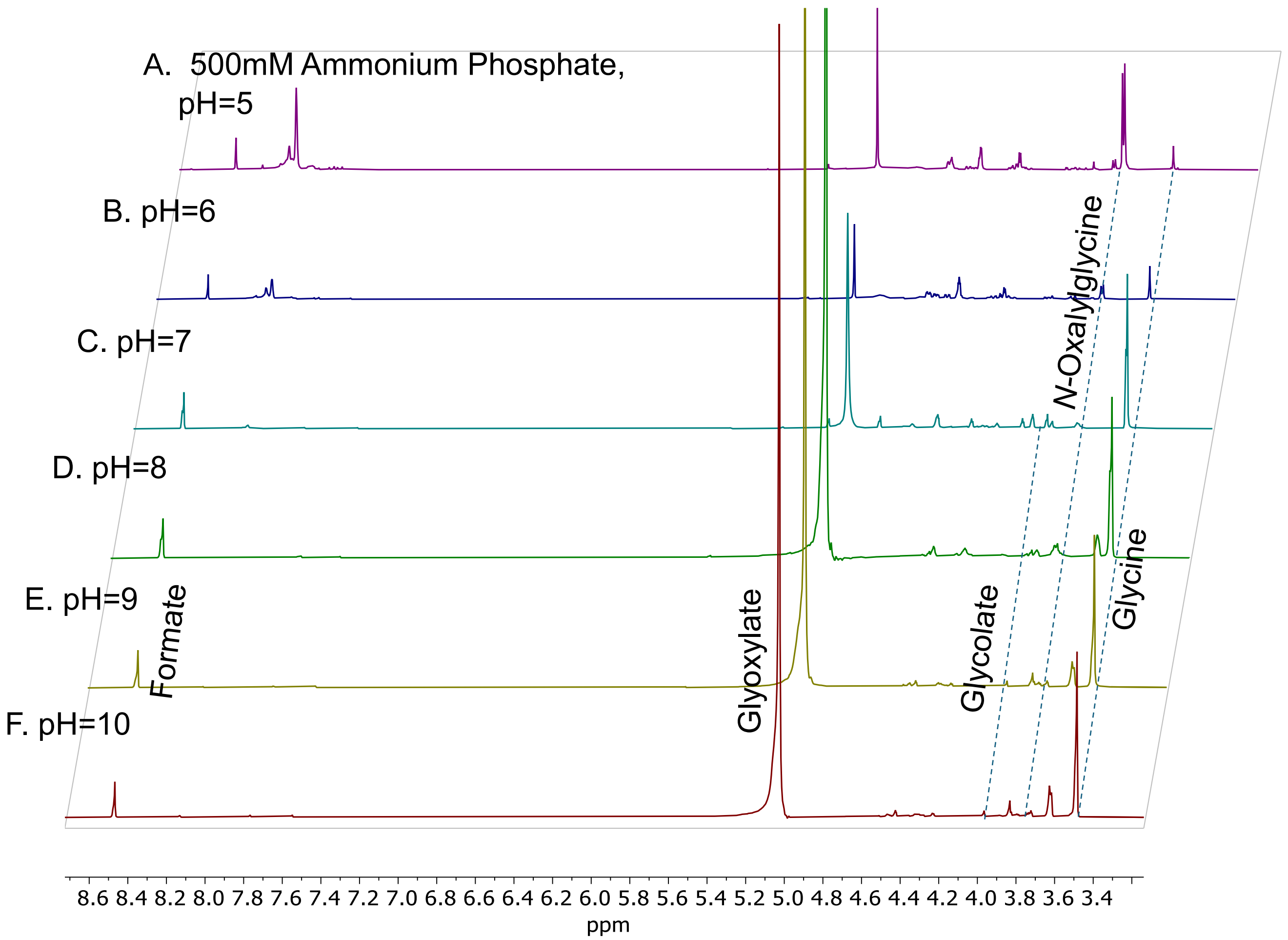

Figure S11. Aza-Cannizzaro reactivity was observed over wide pH range. Glyoxylate (500 mM) in ammonium phosphate (500 mM, pH 5-10) was incubated at 50°C for 48 hr. Reaction aliquot combined with an equal volume of D_2O prior to analysis. Glycine formation was observed at each pH via ^1H NMR-presat (selective pulse at 4.7 ppm to attenuate HOD resonance) (500 MHz; pulse sequence=zgpr30, TSP (3-(trimethylsilyl)propionic-2,2,3,3-d_4 acid, sodium salt) standard). Glycine formation is most favorable above pH 6 (C-F). Other products are observed. Oxalylglycine formation is most prominent at pH 5 (A).

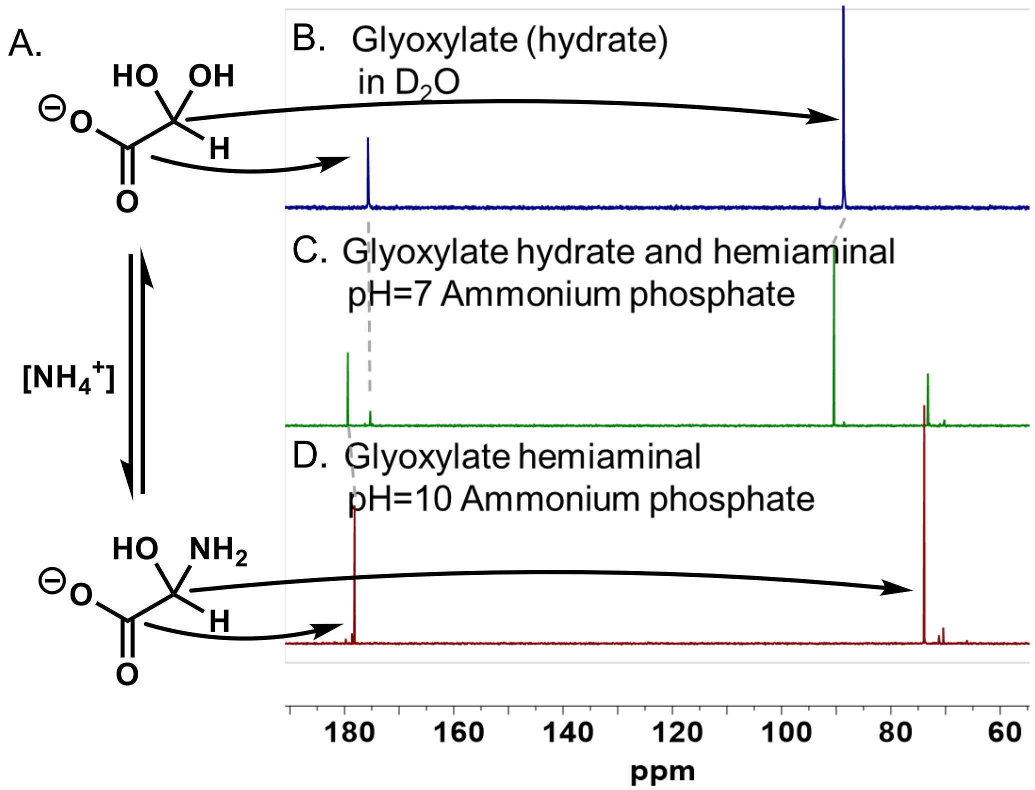

Figure S12. Glyoxylate forms the hemiaminal in the presence of ammonium phosphate. (A) Equilibrium between the hydrate and hemiaminal forms of glyoxylate. (B) In unbuffered D_2O, ^{13}C NMR (125 MHz) indicates that a 500 mM solution of glyoxylate contains mostly the hydrate form (δ175 and 88 ppm). (C) In ammonium phosphate buffer, pH 7, the hemiaminal (δ178 and 73 ppm) is observed in addition to the hydrate. (D) In ammonium phosphate buffer, pH 10, only the hemiaminal is observed.

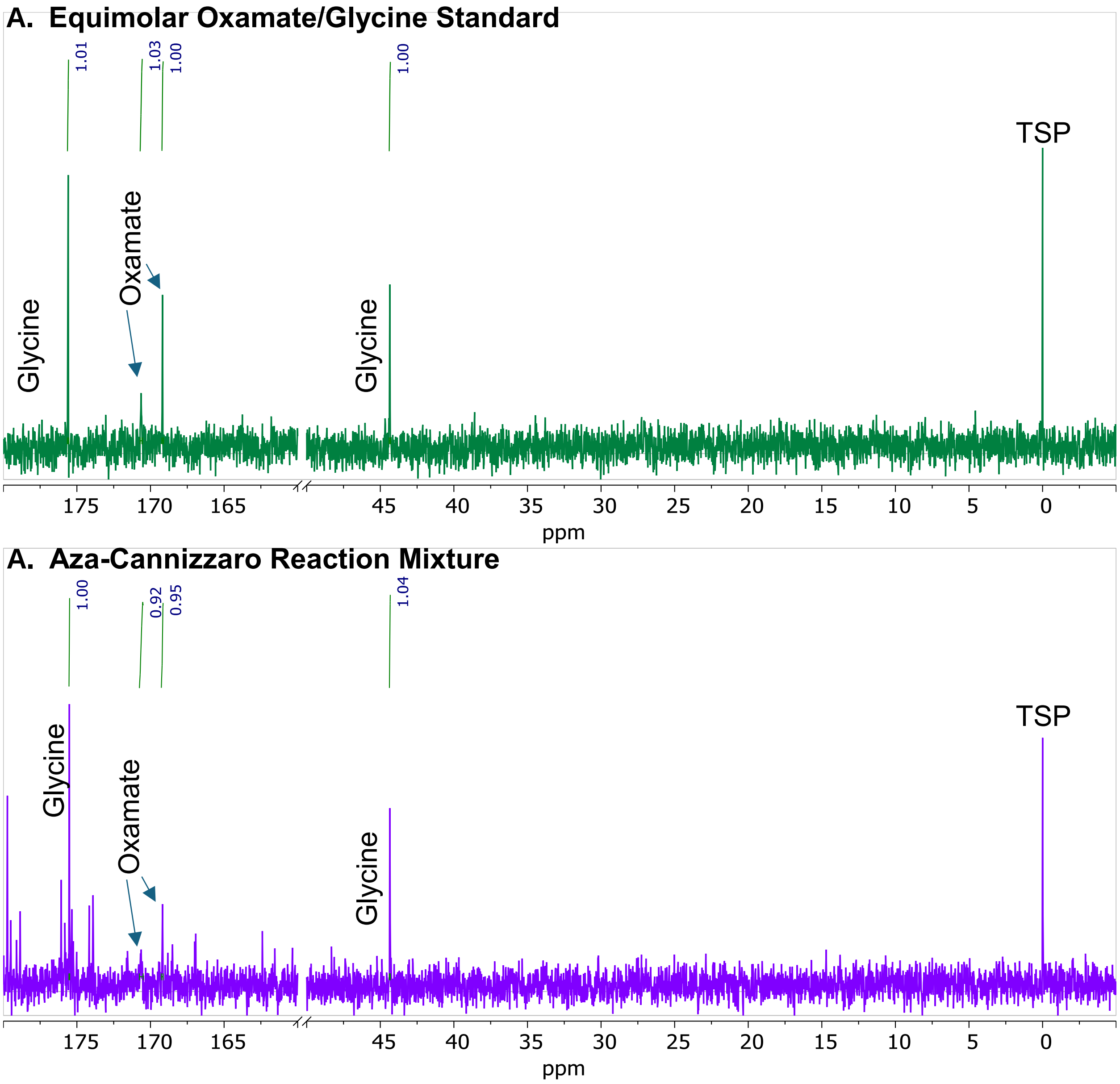

Figure S13. Glycine and oxamic acid are formed in comparable quantities from glyoxylate in ammonium phosphate buffer. Oxamate can be difficult to detect via ^{13}C NMR because (1) neither carbon is bonded to hydrogen, (2) there is significant resonance broadening at pH 7, and (3) the carbon signals have long relaxation times. A zgig30 pulse sequence was used with 20 s relaxation delay and without nOe enhancement to probe whether oxamic acid and glycine were formed in comparable quantities, as predicted by the aza-Cannizzaro mechanistic hypothesis. (A) ^{13}C NMR data for a solution containing equimolar amounts of commercial oxamic acid and glycine in ammonium phosphate buffer, pH 7, mixed with an equal volume D_2O. The ratio of integrals was found to be approximately 1:1. (B) ^{13}C NMR data for a reaction mixture containing 500 mM each of glyoxylate and ammonium phosphate, pH 7, incubated at 50°C for 48 hr. The ratio of integrals was found to be approximately 1:0.9.

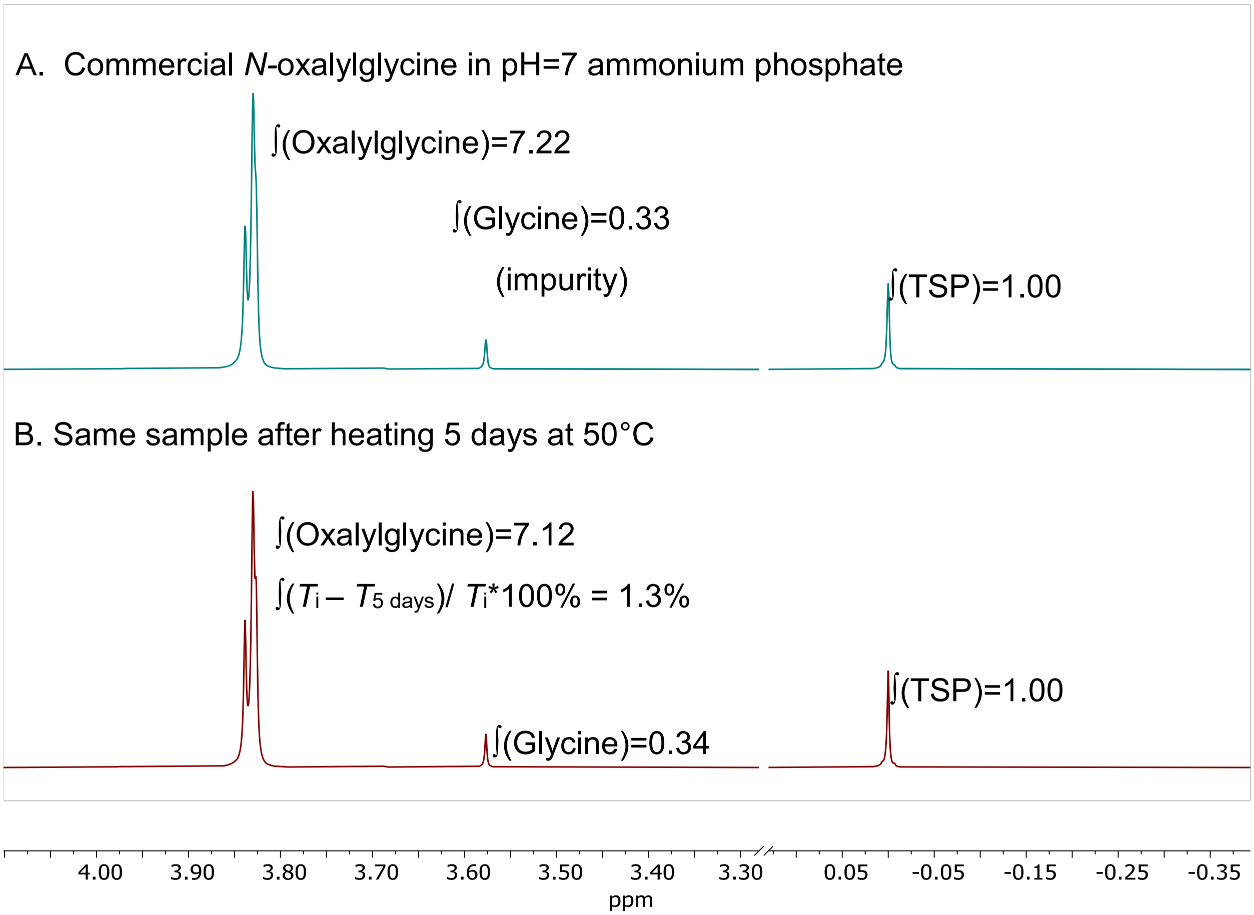

Figure S14. Oxalylglycine is stable to hydrolysis in pH 7 ammonium phosphate buffer. (A) A commercial sample of oxalylglycine was dissolved in 500 mM ammonium phosphate buffer, pH 7. An aliquot was mixed with an equal volume of D_2O containing TSP (3-(trimethylsilyl)propionic-2,2,3,3-d_4 acid, sodium salt) as a standard, and an ^{1}H-presat (selective pulse at 4.7 ppm to attenuate HOD resonance) NMR spectrum (500 MHz) was acquired. (B) The oxalylglycine solution was incubated at 50°C for 5 days, and then another aliquot was analyzed after mixing with an equal volume of D_2O containing TSP. Only 1.3% of the oxalylglycine underwent hydrolysis under these conditions, based on the normalized integral. These findings support our hypothesis that glycine formed upon heating glyoxylic acid in ammonium phosphate buffer arises via an aza-Cannizzaro mechanism, rather than an alternative mechanistic path involving oxalylglycine formation followed by hydrolysis.[5,11]

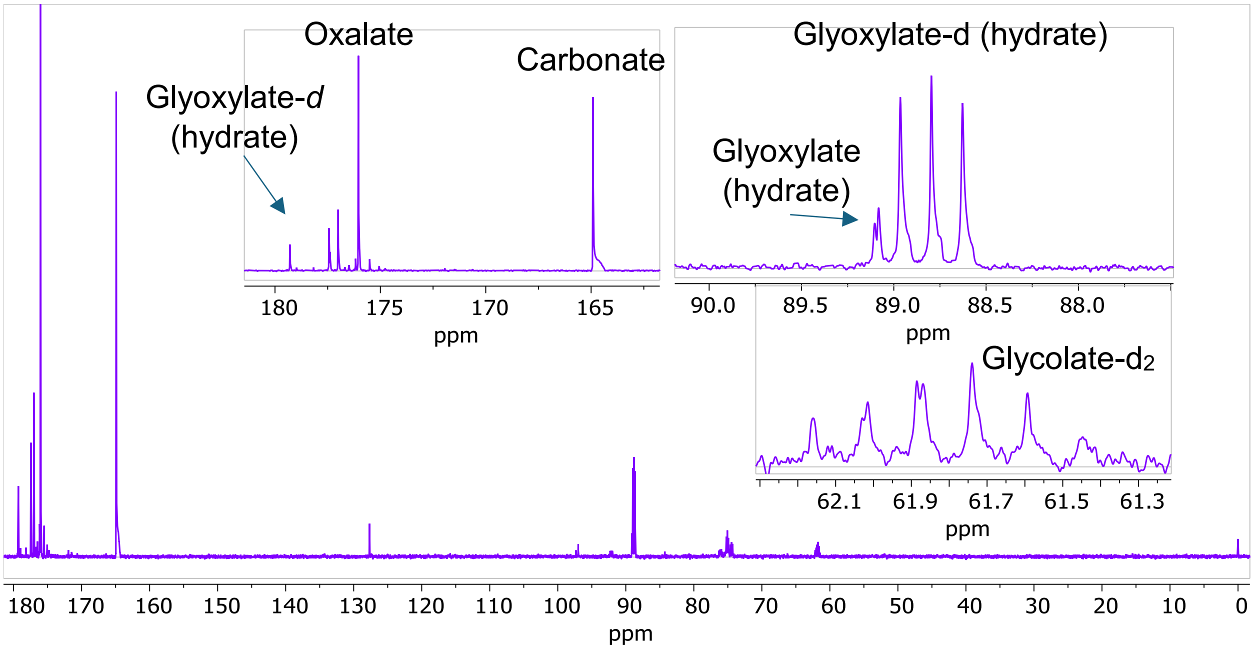

Figure S15. ^{13}C NMR (200 MHz) analysis of glyoxylate-d prepared via magnesium reduction of oxalic acid-d_2. Insets for selected regions are provided. These data show that the sample contains oxalate (unreacted starting material) and glycolate-d_2 (by-product from over-reduction).

A.

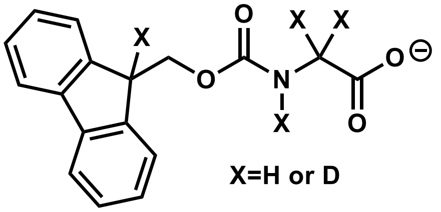

B.

Fmoc-glycine	$[M-H]^-$=296.0928
Fmoc-glycine-d_1	297.0991
Fmoc-glycine-d_2	298.1054
Fmoc-glycine-d_3	299.1117
Fmoc-glycine-d_4	300.1179

C.

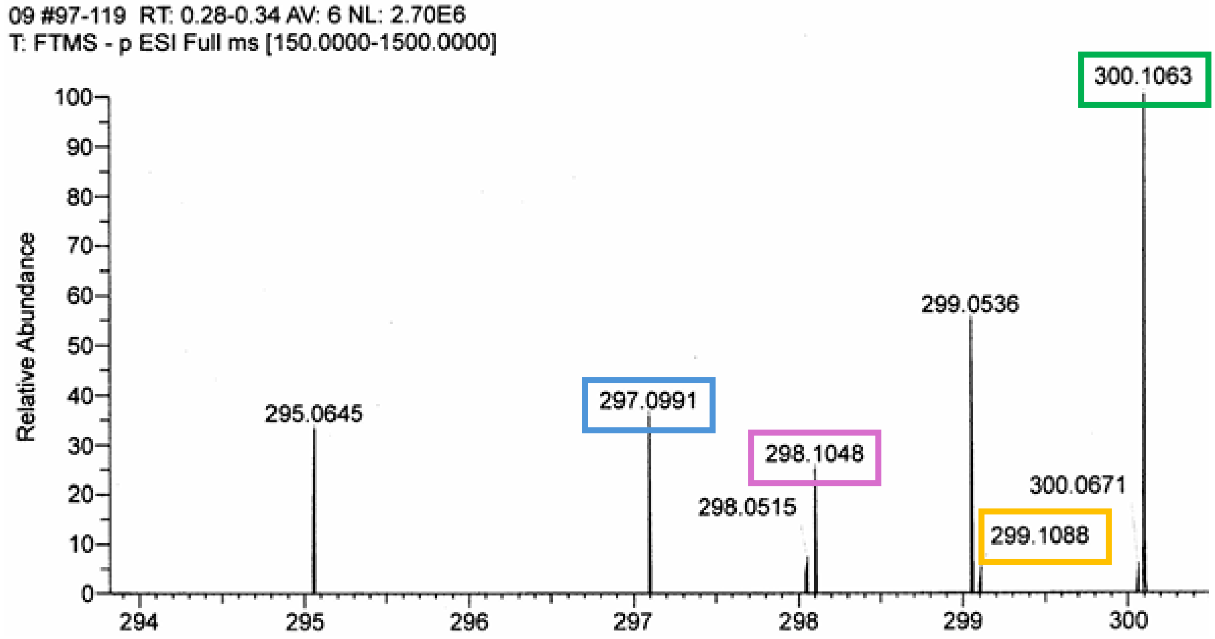

Figure S16. Mass spectrum of Fmoc-glycine-d_x. High resolution ESI M- mass spectrum showing multiple species including Fmoc-glycine-d_x from the aza-Cannizzaro reaction with glyoxylic acid-d as described in the main text. (A) Chemical structure of Fmoc-glycine anion showing four possible positions of deuteration (X). (B) Exact masses calculated for Fmoc-glycine anion contain one, two, three or four deuterium atoms. (C) Fmoc-glycine containing one, two, three or four deuterium atoms detected via mass spectrometry. The species containing zero deuterium atoms was not detected.

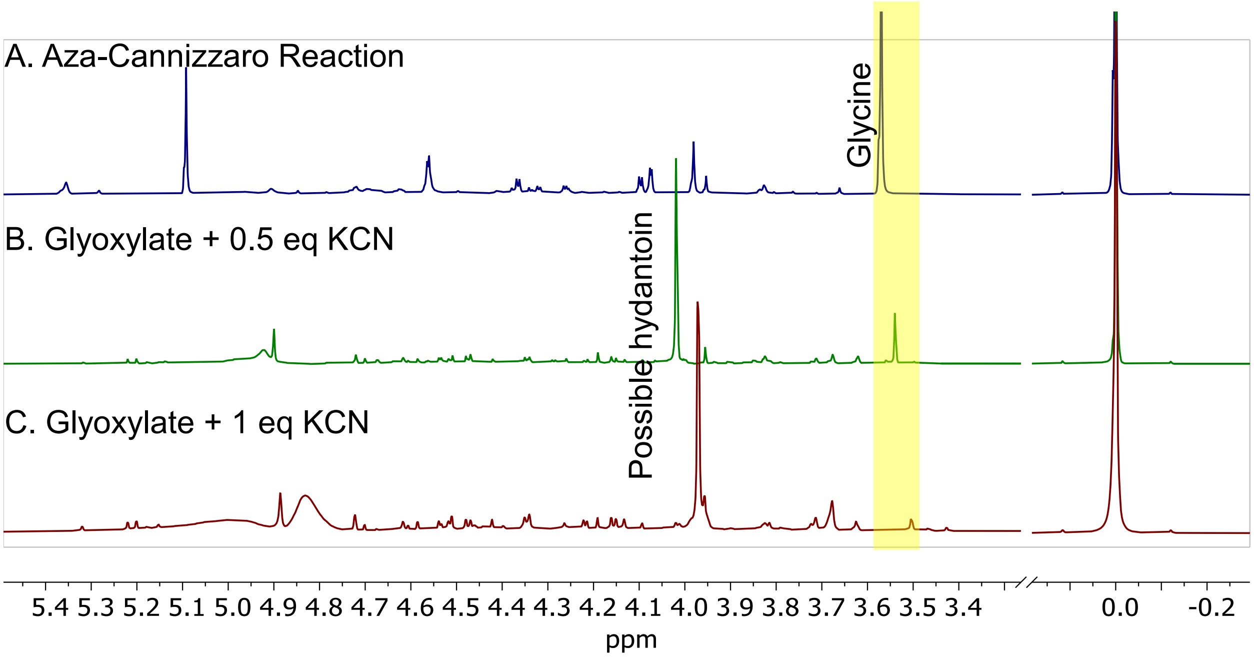

Figure S17. Cyanide inhibits the aza-Cannizzaro reaction. Glyoxylate (500 mM) in ammonium phosphate (500 mM, pH 7) was heated to 50°C for 48 hr with: (A) no additive, (B) 0.5 eq KCN, or (C) 1 eq KCN. Reaction aliquot combined with an equal volume of D_2O prior to analysis. ^{1}H-presat (selective pulse at 4.7 ppm to attenuate HOD resonance) NMR data (500 MHz; pulse sequence=zgpr30, TSP (3-(trimethylsilyl)propionic-2,2,3,3-d_4 acid, sodium salt) standard) show that glycine formation was suppressed in the cyanide-containing solutions.
